# The fatal contribution of serine protease-related genetic variants to COVID-19 outcomes

**DOI:** 10.3389/fimmu.2024.1335963

**Published:** 2024-03-27

**Authors:** Laura Edith Martínez-Gómez, Carlos Martinez-Armenta, Teresa Tusie-Luna, Paola Vázquez-Cárdenas, Rosa P. Vidal-Vázquez, Juan P. Ramírez-Hinojosa, Diana Gómez-Martín, Gilberto Vargas-Alarcón, Rosalinda Posadas-Sánchez, José Manuel Fragoso, Aurora de la Peña, José Manuel Rodríguez-Pérez, Mónica M. Mata-Miranda, Gustavo J. Vázquez-Zapién, Adriana Martínez-Cuazitl, Felipe de J. Martínez-Ruiz, Dulce M. Zayago-Angeles, Luis Ramos-Tavera, Alberto Méndez-Aguilera, María del C. Camacho-Rea, María L. Ordoñez-Sánchez, Yayoi Segura-Kato, Carlos Suarez-Ahedo, Jessel Olea-Torres, Brígida Herrera-López, Carlos Pineda, Gabriela A. Martínez-Nava, Alberto López-Reyes

**Affiliations:** ^1^ Laboratorio de Gerociencias, Dirección General, Departamento de Reconstrucción Articular, Laboratorio Facilitador, Instituto Nacional de Rehabilitación Luis Guillermo Ibarra Ibarra, Secretaría de Salud, Mexico City, Mexico; ^2^ Unidad de Biología Molecular y Medicina Genómica, Instituto Nacional de Ciencias Médicas y Nutrición, Salvador, Zubirán, Mexico City, Mexico; ^3^ Instituto de Investigaciones Biomédicas Universidad Nacional Autónoma de México, Mexico City, Mexico; ^4^ Centro de Innovación Médica Aplicada, Hospital General Dr. Manuel Gea González, Mexico City, Mexico; ^5^ Department of Immunology and Rheumatology, Departamento de Inmunogenética, Departamento de Nutrición Animal, Instituto Nacional de Ciencias Médicas y Nutrición Salvador Zubirán, Secretaría de Salud, Mexico City, Mexico; ^6^ Departamento de Biología Molecular y Endocrinología, Instituto Nacional de Cardiología Ignacio Chávez, Mexico City, Mexico; ^7^ Departamento de Farmacología, Facultad de Medicina, Universidad Nacional Autónoma de México, Mexico City, Mexico; ^8^ Laboratorio de Biología Celular y Tisular, Laboratorio de Embriología, Escuela Médico Militar, Universidad del Ejército y Fuerza Aérea, Mexico City, Mexico; ^9^ Nuevo Hospital General Delegación Regional Sur de la Ciudad de México Instituto de Seguridad y Servicios Sociales de los Trabajadores del Estado (ISSSTE), Mexico City, Mexico

**Keywords:** COVID-19, SERPINE1, TMPRSS2, *Polymorphism*, SARS-CoV-2

## Abstract

**Introduction:**

Serine proteases play a critical role during SARS-CoV-2 infection. Therefore, polymorphisms of transmembrane protease serine 2 (*TMPRSS2*) and serpine family E member 1 (*SERPINE1*) could help to elucidate the contribution of variability to COVID-19 outcomes.

**Methods:**

To evaluate the genetic variants of the genes previously associated with COVID-19 outcomes, we performed a cross-sectional study in which 1536 SARS-CoV-2-positive participants were enrolled. *TMPRSS2* (rs2070788, rs75603675, rs12329760) and *SERPINE1* (rs2227631, rs2227667, rs2070682, rs2227692) were genotyped using the Open Array Platform. The association of polymorphisms with disease outcomes was determined by logistic regression analysis adjusted for covariates (age, sex, hypertension, type 2 diabetes, and obesity).

**Results:**

According to our codominant model, the GA genotype of rs2227667 (OR=0.55; 95% CI = 0.36-0.84; *p*=0.006) and the AG genotype of rs2227667 (OR=0.59; 95% CI = 0.38-0.91; *p*=0.02) of *SERPINE1* played a protective role against disease. However, the rs2227692 T allele and TT genotype *SERPINE1* (OR=1.45; 95% CI = 1.11-1.91; *p*=0.006; OR=2.08; 95% CI = 1.22-3.57; *p*=0.007; respectively) were associated with a decreased risk of death. Similarly, the rs75603675 AA genotype *TMPRSS2* had an OR of 1.97 (95% CI = 1.07-3.6; *p*=0.03) for deceased patients. Finally, the rs2227692 T allele *SERPINE1* was associated with increased D-dimer levels (OR=1.24; 95% CI = 1.03-1.48; *p*=0.02).

**Discussion:**

Our data suggest that the rs75603675 *TMPRSS2* and rs2227692 *SERPINE1* polymorphisms are associated with a poor outcome. Additionally, rs2227692 *SERPINE1* could participate in hypercoagulable conditions in critical COVID-19 patients, and this genetic variant could contribute to the identification of new pharmacological targets and treatment strategies to block the inhibition of TMPRSS2 entry into SARS-CoV-2.

## 1 Introduction

Serine protease cascades control coagulation, and innate immune responses are increased during severe SARS-CoV-2 infection (1, 2). Different enzymes with serine protease activity, such as transmembrane protease serine 2 (TMPRSS2), have been described as critical determinants of spike (S) protein shedding in the SARS-CoV-2 virus and therefore trigger the infection process via the endosomal route or by membrane fusion with the host cell (3, 4). TMPRSS2 expression is crucial for the spread and pathogenesis of SARS-CoV-2. The spike protein of SARS-CoV-2 can be cleaved by circulating thrombotic proteases, thrombin and Factor X, as well as the thrombotic protease plasmin, which contributes to severe COVID-19 complications (1, 5).

The *TMPRSS2* gene is located on chromosome 21q22.3 and comprises 14 exons and 13 introns; its transcriptional activity is controlled by androgen receptors, which play roles in carcinogenesis (6). The enzymatic activity of TMPRSS2 in viral infection and its inhibition have been proposed as novel mechanisms to reduce mortality associated with SARS-CoV-2 infection (7, 8). In this sense, a nonpharmacological strategy to regulate the activity of TMPRSS2 by its endogenous inhibitor plasminogen activator inhibitor-1 (PAI-1) for influenza and coronavirus infections has recently been reported (9).

The serpine family E member 1 (*SERPINE1)* gene encodes plasminogen activator inhibitor-1 (PAI-1), whose principal physiological activity is to inhibit urokinase plasminogen activator (uPA) and tissue-type plasminogen activator (tPA) to further regulate the breakdown of blood clots. Therefore, the modulation of uPA and tPA could control the production of plasmin, D-dimer, and ferritin, which are associated with coagulopathies and adverse outcomes in patients with COVID-19 (10). The presence of D-dimer guarantees that coagulation is amplified and that fibrin deposits are ultimately stabilized (11, 12). SERPINE1 and other serine protease inhibitors have the potential to inhibit TMPRSS2 (5).

The *SERPINE1* gene is located on chromosome 7q22.1, and some polymorphisms in the SERPINE1 promoter region have been associated with severe COVID-19 (13, 14) as well as with a suboptimal fibrinolytic response (15, 16).

Genetic polymorphisms in these genes could modulate genetic predisposition to infection and virus clearance in the host (17). The rs12329760 polymorphism is present in the exonic splicing enhancer site srp40 and could increase the chance of expression due to potential disruption of the exonic splicing enhancer site. rs2070788 and rs75603675 were reported to have higher levels of TMPRSS2 expression and structural changes (13, 17). rs2227631 is located in the promoter and is implicated in PAI regulation. rs5557667 is located in the intronic region between exons 3-4, rs2070682 is located in introns 5-6, and rs222692 is located in introns 7-8 (14, 18).

Given the critical participation of *TMPRSS2* and *SERPINE1* in SARS-CoV-2 infection, it is relevant to investigate whether their genetic variants could be associated with the severity of clinical manifestations and/or fatal outcomes in COVID-19 patients. The aim of this study was to determine the associations of the polymorphisms rs2070788, rs75603675 and rs12329760 of the *TMPRSS2* gene and the polymorphisms rs2227631, rs2227667, rs2070682 and rs2227692 of the *SERPINE1* gene with COVID-19 severity and their relationships with inflammatory biomarkers.

## 2 Materials and methods

### 2.1 Setting and participants

We conducted a cross-sectional study including 1536 patients from different Mexican institutions of governmental health care from June 2020 to March 2021. Nonprobability sampling was performed for unvaccinated patients. The inclusion criteria were as follows: individuals who were not familiar with COVID-19, independent of their sex, were aged ≥18 years with clinical features of COVID-19, and had a positive qRT–PCR test from a nasopharyngeal swab. Participant enrollment was performed in the following public hospitals of the Mexican governmental health system located in Mexico City: Instituto Nacional de Rehabilitación Luis Guillermo Ibarra Ibarra (17/20 AC); Instituto Nacional de Cardiología Ignacio Chávez (20–1202); Hospital Central Militar (045/2020); Instituto Nacional de Ciencias Médicas y Nutrición Salvador Zubirán (REF 3340); and Hospital General Dr. Manuel Gea González (CONBIOETICA09-CEI-024-20161215).

The exclusion criteria were pregnancy and incomplete clinical records. The participants were classified according to disease severity as previously described (19). All demographic and clinical data were obtained from the clinical records of each included patient.

This research complied with the Declaration of Helsinki and was approved by the participating health institutions’ ethics and research committees. In addition, all participants provided written informed consent before agreeing to participate in the study.

### 2.2 Blood, serum, and DNA sample processing

Peripheral blood samples were collected from each participant at the hospital’s triage for DNA and serum isolation. Genomic DNA was isolated using a specialized commercial kit (QIAmp DNA Blood Mini Kit, part number 51106, Qiagen, Hilden, Germany). The quality of the DNA samples was evaluated by the 260/280 nm absorbance ratio, and 1% agarose gels were stained with SYBR^®^ Green (Invitrogen, CA, USA). Then, the DNA concentration was quantified using a Thermo Scientific NanoDrop spectrophotometer to measure the absorbance at wavelengths ranging from 260 to 280 nm; the quality of the samples ranged from 1.8-2.0, and the concentration was adjusted to 20 ng/μl. In addition, a vacutainer tube with SST II Advance gel was used for serum isolation. Serum samples were separated and stored at -80°C until further use.

### 2.3 Selection of single nucleotide polymorphisms (SNPs)

The polymorphisms of *TMPRSS2* (rs2070788, rs75603675, rs12329760) and *SERPINE1* (rs2227631, rs2227667, rs2070682, rs2227692) were selected on the basis of their previous scientific evidence of associations with different diseases in any population that included independent genetic studies from 2003–2020. The included polymorphisms had to present a minor allele frequency (MAF) ≥5%, according to the 1000 Genomes Project or Hap map in the Mexican population (MXL) or the Iberia (IBS) population (20).

For genotyping, 10 ng/μl genomic DNA was transferred into genotyping OpenArray plates, which previously contained the specific genotyping primers and probes, using the AccuFill system. Real-time PCR amplification was performed according to the supplier’s protocol using the Open Array Platform through a Quant Studio 12 K Flex System (Thermo Fisher Scientific, Waltham, MA, USA), and the results were analyzed using TaqMan Genotyper v1.6 software.

### 2.4 Statistical analysis

We performed an exploratory bivariate analysis. Nonparametric variables are reported as medians (p50) with interquartile ranges (IQRs). We used the Kruskal−Wallis test for continuous variables, while categorical variables were evaluated with the chi-square test. Hardy−Weinberg equilibrium was tested for all SNPs with a mild outcome. Linkage disequilibrium estimations between SNPs and haplotypes were performed with Haploview V4.2 Software (Broad Institute of Massachusetts Institute of Technology and Harvard University, Cambridge, MA, USA).

Binary logistic regression analysis was applied to determine the genetic associations with the outcomes of patients with COVID-19. The main inherence models were considered and adjusted for risk confounding variables such as age, sex, obesity, type 2 diabetes, and hypertension. In addition, the final models were assessed using the Hosmer−Lemeshow goodness-of-fit test.

To determine the association of *SERPINE1* polymorphisms with increased D-dimer levels, we conducted a logistic analysis adjusted for age, sex, obesity, type 2 diabetes, and hypertension, stratifying D-dimer according to serum levels. The cutoff points for high and low concentrations were ≥500 ng/mL and <500 ng/mL, respectively (21).

The statistical analysis used STATA v.16 (StataCorp, Texas, USA). A P value < 0.05 indicated statistical significance.

## 3 Results

### 3.1 Patients and clinical traits

We enrolled 1,728 patients; however, subjects with incomplete data were excluded (n=172). For the final analysis, 1,536 patients were classified according to disease severity into mild (35%), severe (33%), critical (18%) and deceased (14%) groups. Sixty-four percent of the total population were males, and the median age was 55 years (IQR=45-65). However, the median age of the deceased group was 67 years (IQR=57-75.5). Further relevant clinical and laboratory features are depicted in **Table 1**.

**Table 1:** Clinical parameters and anthropometric characteristics of the population.

	Total n = 1,536 (100%)	Mild n = 543 (35%)	Severe n = 503 (33%)	Critical n = 278 (18%)	Deceased n = 212 (14%)	P value
Age (years)*	55 (45-65)	51 (39-63)	54 (45-63)	56 (47-64)	67 (57-75.5)	**<0.001**
Sex Male**	979 (64%)	316 (58%)	329 (65%)	191 (69%)	143 (67%)	**0.007**
Type 2 diabetes**	478 (31%)	124 (23%)	167 (33%)	99 (36%)	88 (41%)	**<0.001**
Obesity**	568 (37%)	203 (37%)	195 (38%)	113 (41%)	57 (27%)	**0.009**
Hypertension**	506 (33%)	141 (26%)	162 (32%)	96 (34%)	107 (50%)	**<0.001**
D-Dimer (ng/mL)*	593.5 (283-1016)	586 (330.5-951.5)	609 (301-1070)	492 (87-951)	691.4 (238-1242)	**0.02**
Ferritin (ng/mL)*	503.8 (253.9-913.3)	385 (165-724)	521.2 (266.2-955.1)	572.2 (376.2-1000.8)	692.7 (361.7-1067.4)	**<0.001**
LDH (U/L)*	300.5 (212.1-427)	151 (122-190)	282.1 (221-372)	391.8 (280-482)	407 (317-484.8)	**<0.001**
Platelets Millions/mm^3^*	234.5 (178.5-303.5)	222 (182-264)	237 (181-319)	245 (186-322)	216 (164-290)	**0.01**
C-Reactive Protein (mg/L)*	16.34 (5.41-36.3)	2.8 (1.1-10.2)	17.3 (6.5-71.5)	19.1 (8.7-33.1)	22.1 (14.4-41.9)	**<0.001**

*Kruskal−Wallis test; **Chi−square test. The value in bold denotes statistical significance.

Our data revealed that the highest levels of D-dimer (p50 = 691.41 ng/mL (IQR=238-1242)), ferritin (p50 = 692.7 ng/mL (IQR=361.7-1067.4)), LDH (p50 = 407 ng/mL (IQR=317-484.8)) and C-reactive protein (p50 = 22.1 mg/L (IQR=14.4-41.9)) were detected in the deceased group (**Table 1**). Although the laboratory parameters tended to increase with disease severity, this was not the case for D-dimer in the critical group.

### 3.2 Genotypes and allelic frequencies by disease severity and linkage disequilibrium

The genotypic and allelic frequencies among the COVID-19 groups were assessed for each *TMPRSS2* (rs2070788, rs75603675, rs12329760) and *SERPINE1* (rs2227631, rs2227667, rs2070682, rs2227692) polymorphism (**Supplementary 1**). We identified two genetic variants out of seven with a statistically significant difference in allelic frequency distribution among COVID-19 patients, corresponding to rs2070788 (P=0.003) and rs7560375 (P=0.04) of the *TMPRSS2* gene. Nevertheless, only rs2070788 was also significant for genotype distribution (P=0.009).

**Figure 1:**
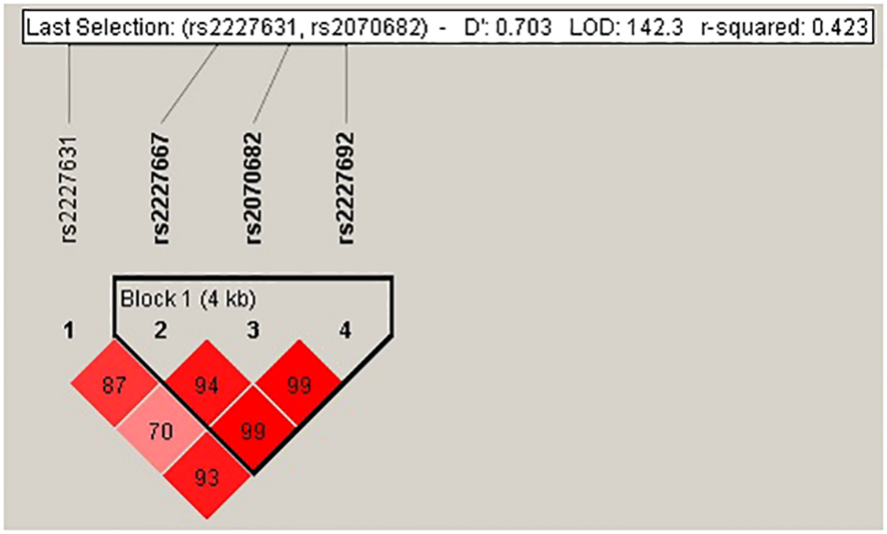
Linkage disequilibrium of rs2227631, rs2227667, rs2070682 and rs2227692 of the *SERPINE1* gene. The D´ value showed a LD of rs2227667, rs2070682 and rs2227692.

Our data showed that only the *TMPRSS2* rs7560367*5* and *SERPINE1* rs2227667 genotypes were not in Hardy−Weinberg equilibrium (P=0.03 and P=0.02, respectively). The *SERPINE1* polymorphisms rs2227667, rs2070682 and rs2227692 displayed linkage disequilibrium (LD), with D´ values of 0.99 and r^2 = ^0.74 (**Figure 1**). The frequencies of the haplotypes were 27% for ATC, 27% for ATT, 13% for GTC, 13% for GCC, 10% for GTT and 10% for GCT. The *TMPRSS2* polymorphisms did not show an LD.

### 3.3 Logistic regression analysis of *TMPRSS2* and *SERPINE1* polymorphisms in COVID-19 patients

Logistic regression analysis adjusted for age, sex, and comorbidities (hypertension, type 2 diabetes, and obesity) revealed a statistically significant association between the *TMPRSS*2 rs2070788 and rs75603675 genetic variants and between the *SERPINE1* rs2227631, rs222667 and rs2227692 and critical and deceased outcomes. **Table 2** summarizes the significance of each *TMPRSS*2 and *SERPINE1* polymorphism with respect to COVID-19 outcomes.

**Table 2:** Associations of *TMPRSS2* and *SERPINE1* polymorphisms with COVID-19 severity.

Polymorphism	Severe			Critical			Deceased		
	OR*	95% CI	P	OR*	95% CI	P	OR*	95% CI	P
*TMPRSS2*									
*rs2070788*									
G	1			1			1		
A	0.86	0.72-1.03	0.12	**0.76**	**0.61-0.94**	**0.02**	1.18	0.91-1.53	0.2
GG^c^	1			1			1		
GA^c^	0.84	0.66-1.17	0.40	0.72	0.51-1.00	0.05	1.04	0.67-1.61	0.85
AA^c^	0.73	0.51-1.07	0.11	0.64	0.41-0.99	0.05	1.38	0.84-2.3	0.21
GA+AA^d^	0.84	0.64-1.11	0.22	**0.69**	**0.51-0.95**	**0.03**	1.14	0.76-1.72	0.51
AA^r^	0.79	0.57-1.11	0.18	0.77	0.52-1.15	0.20	1.13	0.88-2.07	0.17
*rs75603675*									
C	1			1			1		
A	0.88	0.72-1.08	0.25	0.88	0.68-1.13	0.33	1.27	0.96-1.69	0.08
CC^c^	1			1			1		
CA^c^	0.82	0.62-1.08	0.17	0.85	0.61-1.18	0.34	1.04	0.71-1.54	0.83
AA^c^	0.88	0.56-1.4	0.60	0.86	0.49-1.52	0.61	**1.97**	**1.07-3.6**	**0.03**
CA+AA^d^	0.83	0.65-1.08	0.17	0.85	0.62-1.16	0.32	1.19	0.83-1.72	0.34
AA^r^	0.95	0.61-1.48	0.82	0.92	0.53-1.60	0.77	**1.93**	**1.07-3.47**	**0.03**
*rs12329760*									
C	1			1			1		
T	0.88	0.67-1.16	0.39	0.85	0.62-1.18	0.34	1.19	0.83-1.71	0.32
CC^c^	1			1			1		
CT^c^	0.86	0.62-1.18	0.36	0.83	0.57-1.21	0.35	1.30	0.85-1.99	0.22
TT^c^	0.92	0.37-2.26	0.85	1	0.42-2.8	0.87	1.03	0.33-3.18	0.96
CT+TT^d^	0.86	0.64-1.18	0.36	0.86	0.60-1.23	0.41	1.27	0.85-1.92	0.25
TT^r^	0.95	0.39-2.33	0.92	1.12	0.43-2.91	0.80	0.96	0.32-2.94	0.95
*SERPINE1*									
*rs2227631*									
G	1			1			1		
A	0.89	0.72-1.11	0.32	0.89	0.69-1.16	0.42	0.79	0.58-1.09	0.15
GG^c^	1			1			1		
GA^c^	0.92	0.69-1.23	0.60	0.86	0.62-1.21	0.39	**0.55**	**0.36-0.84**	**0.006**
AA^c^	0.76	0.44-1.31	0.33	0.87	0.47-1.61	0.69	1.09	0.55-2.18	0.79
GA+AA^d^	0.89	0.68-1.17	0.43	0.86	0.62-1.19	0.38	**0.64**	**0.43-0.94**	**0.02**
AA^r^	0.79	0.46-1.33	0.38	0.93	0.51-1.68	0.80	1.35	0.69-2.64	0.38
*rs2227667*									
A	1			1			1		
G	0.92	0.76-1.12	0.44	1.04	0.79-1.35	0.78	**0.73**	**0.56-0.96**	**0.03**
AA^c^	1			1			1		
AG^c^	1.13	0.82-1.55	0.42	1.27	0.87-1.84	0.21	**0.59**	**0.38-0.91**	**0.02**
GG^c^	0.83	0.56-1.22	0.34	1.00	0.63-1.56	1	0.60	0.35-1.01	0.06
AG+GG^d^	1.03	0.76-1.38	0.84	1.17	0.83-1.67	0.36	**0.59**	**0.39-0.89**	**0.01**
GG^r^	0.76	0.54-1.07	0.12	0.86	0.59-1.26	0.44	0.81	0.51-1.29	0.38
*rs2070682*									
T	1			1			1		
C	1	0.79-1.27	0.97	1.04	0.79-1.35	0.78	0.96	0.70-1.32	0.82
TT^c^	1			1			1		
TC^c^	1.01	0.76-1.35	0.94	1.03	0.73-1.44	0.86	0.65	0.42-0.99	0.05
CC^c^	0.98	0.50-1-94	0.97	1.11	0.52-2.36	0.79	1.77	0.83-3.80	0.14
TC+CC^d^	1.00	0.76-1.33	0.95	1.03	0.75-1.43	0.82	0.77	0.52-1.16	0.22
CC^r^	0.98	0.50-1.92	0.96	1.09	0.51-2.31	0.81	2.04	0.97-4.31	0.06
*rs2227692*									
C	1			1			1		
T	1.10	0.90-1.33	0.33	0.97	0.78-1.22	0.84	**1.45**	**1.11-1.91**	**0.006**
CC^c^	1			1			1		
CT^c^	1.16	0.84-1.60	0.35	1.04	0.72-1.51	0.82	1.41	0.42-0.99	0.15
TT^c^	1.19	0.82-1.73	0.35	0.95	0.61-1.47	0.81	**2.08**	**1.22-3.57**	**0.007**
CT+TT^d^	1.17	0.86-1.58	0.29	1.01	0.72-1.42	0.95	**1.60**	**1.03-2.50**	**0.03**
TT^r^	1.08	0.79-1.50	0.61	0.92	0.63-1.35	0.68	**1.67**	**1.07-2.59**	**0.02**

*Adjusted by age, sex, hypertension status, type 2 diabetes status and obesity status. Inheritance models= c, codominant; d, dominant; r, recessive. The value in bold denotes statistical significance.

### 3.4 Polymorphisms associated with protection

The protective effects of the *TMPRSS2* and *SERPINE1* genetic variants are shown in **Table 2**. For rs2070788, the A allele had an OR of 0.76 (95% CI = 0.61-0.94; P=0.02), indicating a critical COVID-19 outcome. Moreover, the dominant model (GA+AA) exerted a protective effect, with an OR of 0.69 (95% CI 0.51-0.95; P=0.03) for critical outcomes.

Interestingly, patients who died from COVID-19 were the main group in which rs2227631 and rs2227667 of *SERPINE1* showed a significant protective effect. Similarly, the rs2227631 GA genotype had an OR of 0.55 (95% CI = 0.36-0.84; P=0.006), and similar results were found for the dominant model (AG+GG), with an OR of 0.64 (95% CI = 0.43-0.94; P=0.02). Regarding the rs2227667 G allele, a significant protective association was also observed with decreased COVID-19 incidence (OR=0.73; 95% CI = 0.65-0.96; P=0.03). Similar results were obtained for the GA genotype, with an OR of 0.59 (95% CI = 0.38-0.91; P=0.02) in the deceased group.

### 3.5 Risk polymorphisms

According to the main inherent genetics models of *TMPRSS*2 rs75603675, a statistically significant association was found between the AA genotype and decreased COVID-19 incidence (OR=1.97; 95% CI 1.07-3.6; P=0.03). Similarly, a significant association was observed for the rs2227692 T allele of the *SERPINE1* gene, with the deceased outcome showing an OR of 1.45 (95% CI = 1.11-1.91; P=0.006). The main inherence genetics models were associated with the deceased group (**Table 2**). Interestingly, the codominant model for the TT genotype had an OR of 2.08 (95% CI=1.22-3.57; P=0.007) for the deceased outcome, while the dominant (TC+TT) and recessive models had ORs of 1.60 (95% CI=1.03-2.50; P=0.03) and 1.67 (95% CI=1.07-2.59; P=0.02), respectively.

We performed a logistic regression of *SERPINE1* haplotypes and only found a statistically significant association between the ATT haplotype and the deceased group, with an OR of 1.6 (95% CI = 1.08-2.25; P=0.02).

### 3.6 Associations of *TMPRSS2* and *SERPINE1* polymorphisms with clinical laboratory parameters stratified by outcome

To understand the impact of each polymorphism on the immune-hematological response to SARS-CoV-2, we further studied the relationships of each polymorphism with diverse laboratory features among COVID-19 patients. In this sense, the analysis revealed a significant difference between the minor allele carriers of *SERPINE1* rs2227667, which had a decreased LDH concentration compared to the major allele (p50 = 306 U/L; IQR=205-431) vs. p50 = 322 U/L; IQR=222-453, respectively) (P=0.03). Although the minor allele of rs2227631 also tended to decrease LDH concentration, there was no statistically significant difference from the major allele. On the other hand, the risk variant of the *SERPINE*1 gene (rs2227692) had increased D-dimer levels compared with those of the minor and major alleles (p50 = 613.5 ng/mL; IQR=281.6-1035 vs. p50 = 545.8 ng/mL; IQR=229-1001, respectively) (P=0.02). This was also observed for the LDH concentration (p50 = 317 U/L; IQR=222-453 vs. p50 = 294 U/L; IQR=202-426.1) (P=0.01) (**Table 3**).

**Table 3:** Laboratory parameters of COVID-19 patients stratified by polymorphism alleles.

*TMPRSS2*				
	Total	Allele	Allele	P value*
*rs2070788*		G	A	
Platelets	234.5 (178.5-303.5)	238 (184-305)	224 (175-298)	0.06
Ferritin (ng/mL)	503.5 (253.6-910.6)	508.05 (254-916.9)	491.8 (253-898.7)	0.34
D Dimer (ng/mL)	592 (278-1013)	585 (272.6-1005)	592 (284-1022)	0.45
C-Reactive Protein (mg/L)	16.34 (5.41-36.3)	16.37 (6-35.24)	16.13 (5-36.58)	0.49
LDH (U/L)	300.5 (212.1-427)	312 (214-430)	290 (211-424)	0.29
*rs75603675*		C	A	
Platelets	235 (178-304)	235 (181-303)	232 (174-304)	0.92
Ferritin (ng/mL)	500.4 (253.1-900)	504.5 (253-916.9)	486.6 (253.1-841.5)	0.31
D Dimer (ng/mL)	593.5 (281-1013.5)	595.5 (281-1035)	591 (274.5-983.5)	0.24
C-Reactive Protein (mg/L)	16.31 (5.375-39.005)	16.435 (5.63-49.2)	15.06 (4.5-34)	0.05
LDH (U/L)	299 (211-426)	306.61 (214-426)	288.8 (204.8-428)	0.25
*rs12329760*		C	T	
Platelets	237 (183-308)	237 (184-307)	236 (172-312)	0.44
Ferritin (ng/mL)	491.8 (245.3-896.4)	492.1 (253-896.4)	482.5 (220.6-889.8)	0.52
D Dimer (ng/mL)	564 (251-1002.41)	567 (252-1006)	528 (241-951)	0.48
C-Reactive Protein (mg/L)	18.215 (5.2-64.1)	18.38 (5.42-67.5)	16.91 (2.8-55.5)	0.24
**LDH (U/L)**	**299.5 (207-435.25)**	**307 (214-438)**	**256 (179.05-426)**	**0.02**

*SERPINE1*				
*rs2227631*		G	A	
Platelets	237 (181.5-307.5)	235 (178-303)	238 (186-324)	0.23
Ferritin (ng/mL)	491.8 (243.9-898.7)	491.8 (242.6-904.3)	487.5 (245.3-886)	0.70
D Dimer (ng/mL)	568 (260-1005)	579 (267-1006)	524 (208-002.41)	0.10
C-Reactive Protein (mg/L)	18.215 (5.26-69.7)	18.38 (5.47-72.6)	17.565 (4.66-49.9)	0.28
LDH (U/L)	299 (205-436.5)	307.305 (214-440)	278.45 (188-421)	0.06
*rs2227667*		A	G	
Platelets	239 (181-309)	238 (179-309)	242 (184-310)	0.69
Ferritin (ng/mL)	499.9 (257-910.6)	510.1 (258.9-940)	491.9 (253.6-842.5)	0.19
D Dimer (ng/mL)	585 (265-1018)	611 (281.6-1035)	563 (234-1008)	0.09
C-Reactive Protein (mg/L)	19.145 (5.83-75.85)	19.98 (6.5-77.8)	18.3 (5-73.9)	0.27
**LDH (U/L)**	**313.8 (217-445)**	**322 (222-453)**	**306 (205-431)**	**0.03**
*rs2070682*		T	C	
Platelets	238 (181-309)	238 (180-305)	238 (183-324)	0.47
Ferritin (ng/mL)	495.6 (255.4-907.4)	492.3 (253.3-907.4)	504.1 (268.6-907.4)	0.50
D Dimer (ng/mL)	581 (265-1016)	592 (276-1005)	577 (200-1057)	0.44
C-Reactive Protein (mg/L)	19.08 (5.76-73.9)	18.92(5.9-71.6)	20.165 (5.42-90)	0.47
LDH (U/L)	312.5 (216.1-442.3)	312 (217-447)	313 (213.3-426.1)	0.65
*rs2227692*		C	T	
Platelets	237 (181-309)	238 (185-308)	235 (177-312)	0.62
Ferritin (ng/mL)	492.5 (253.6-900)	491.8 (252.1-864.7)	507.6 (257-917.9)	0.31
**D Dimer (ng/mL)**	**577 (260-1008)**	**545.875 (229-1001)**	**613.5 (281.6-1035)**	**0.02**
C-Reactive Protein (mg/L)	19.05 (5.76-71.62)	17.6 (5-71.62)	19.755 (6.945-71.905)	0.19
**LDH (U/L)**	**307.5 (212.2-439.5)**	**294 (202-426.1)**	**317 (222-453)**	**0.01**

*Kruskal−Wallis test. The value in bold denotes statistical significance.

Due to the possible relationship between D-dimer and *SERPINE1* gene risk polymorphisms, we further evaluated the association of each genetic variant with D-dimer, which was dichotomized based on the cutoff value of 500 ng/mL. Our population study revealed a significant association between the T allele of the *rs2227692* polymorphism and a high D-dimer concentration (>500 ng/mL) (OR=1.24; 95% CI=1.03-1.48; P=0.02, **Table 4**). Interestingly, when COVID-19 outcomes were stratified, rs2227692 maintained its association in the deceased patient group (OR=2.04; 95% CI=1.23-3.37; p=0.006) (**Table 5**).

**Table 4:** Association of high D-dimer concentrations with *SERPINE1* risk polymorphisms.

SERPINE1 SNPs	D-dimer (>500 ng/mL)		
*rs2227631*	OR*	95% CI	P
G	1		
A	0.85	0.69-1.05	0.14
* rs2227667*			
A	1		
G	0.83	0.69-1.00	0.05
* rs2070682*			
T	1		
C	0.84	0.67-1.05	0.13
* rs2227692*			
C	**1**		
T	**1.24**	**1.03-1.48**	**0.02**

*Adjusted by age, sex, hypertension status, type 2 diabetes status and obesity status. The value in bold denotes statistical significance.

**Table 5:** Laboratory parameters of COVID-19 patients stratified by polymorphism alleles.

*SERPINE1* *SNPs*	Mild			Severe			Critical			Deceased		
	OR*	95% CI	P	OR*	95% CI	P	OR*	95% CI	P	OR*	95% CI	P
*rs2227631*												
Low	1			1			1			1		
High	0.91	0.63-1.31	0.62	0.82	0.56-1.20	0.32	0.83	0.43-1.31	0.44	0.74	0.41-1.29	0.28
* rs2227667*												
Low	1			1			1			1		
High	0.87	0.62-1.23	0.45	0.77	0.56-1.07	0.13	0.89	0.61-1.31	0.58	0.61	0.37-1.01	0.06
* rs2070682*												
Low	1			1			1			1		
High	0.93	0.61-1.40	0.72	0.94	0.63-1.40	0.78	**0.59**	**0.37-0.94**	**0.03**	0.78	0.44-1.39	0.41
* rs2227692*												
Low	1			1			1			1		
High	1.14	0.82-1.58	0.42	1.28	0.93-1.78	0.13	1.16	0.79-1.71	0.44	**2.04**	**1.23-3.37**	**0.006**

*Adjusted by age, sex, hypertension status, type 2 diabetes status and obesity status. The value in bold denotes statistical significance.

## 4 Discussion

Since COVID-19 emerged, people infected with SARS-CoV-2 have experienced different clinical outcomes. Nevertheless, the development of fatal COVID-19 cases has been strongly associated with comorbidities, including obesity, type 2 diabetes, and cardiovascular diseases, which can exacerbate the inflammatory state (21–25).

In COVID-19 pathogenesis, many factors contribute to viral pathogenesis. Several authors have described three points: (1) recognition of the virus by cellular receptors; (2) suppression of the antiviral response; and (3) the ability to evade the immune system (23, 26).

SARS-CoV-2 uses the ACE2 receptor and TMPRSS2 to promote cellular entry by cleaving the S protein into S1 and S2 (27–29). TMPRSS2 is used by diverse viruses to infect humans, and it has been associated with physiological processes such as digestion, tissue remodeling, blood coagulation, fertility, inflammatory responses, and pain, among others, and the expression of TMPRSS2 is regulated by aging (30). Recent studies have explained the possible role of structural and regulatory variants of *TMPRSS2* in susceptibility to COVID-19. In this sense, the variants p.Gly8Val/c.23G>T (rs75603675) and p.Val197Met/c.589G>A (rs12329760) have been reported to influence its interaction with ACE2 and the S protein (29, 31, 32). rs2070788 has been reported to be highly expressed in the lungs of patients at risk of developing severe COVID-19 (33), suggesting that these variants could play an important role in the severity of SARS-CoV-2 infection (34–36). Nevertheless, the study of *TMPRSS2* polymorphisms has been described in some populations with contradictory results (37–39).

Our study explored the association of *TMPRSS*2 genetic variants with COVID-19 severity; interestingly, we observed that rs75603675 increased the risk of death due to COVID-19. *rs75603675* is a missense variant, c.23G>T, which modifies protein structure to decrease specificity or induce impaired interaction with viral proteins. The isoforms of TMPRSS2 are composed of 492 amino acids and 22 cysteine residues (40, 41). Some reports have described different strains of SARS-CoV-2 with the TMPRSS2 receptor; of the different SARS-CoV-2 variants, the omicron is the least dependent on TMPRSS2 (42). In the present study, we did not characterize the viral strain; however, according to epidemiological data reported in Mexico from the first, second, and third waves (summer 2020-summer 2021), the SARS-CoV-2 variants were alpha-gamma, which is associated with a high mortality rate (43). In this sense, Sabyasachi Senapati et al. reported that rs75603675 could disrupt the local protein structure, increasing the stability of TMPRSS2, while rs12329760 increased the number of S protein domains (31). However, other SNPs in *TMPRSS2* influence its expression (29). According to these findings, using a molecular docking approach, Sabayasachi Senapati et al. identified some phytochemicals that could bind to TMPRSS2 during host−interactions. However, the authors recommended *in silico* and *in vitro* studies to validate the efficacy of these phytochemicals.

The rs75603675 23G>T (Gly8Val) and rs12329760 (589 G>A, Val197Met) are theoretically considered to be responsible for changes in the interaction of TMPRSS2 with the S protein of the virus (26).

Posadas-Sánchez et al. previously studied the associations of the *TMPRSS2* rs462574, rs456298, rs2298659, and rs12329760 (pV197M) polymorphisms with the risk of infection with SARS-CoV-2 in a Mexican population and reported that only rs462574 and rs456298 were associated with this association (44). Consistent with our results, rs12329760 (pV197M) does not show an association with either the risk or protection of infection. rs12329760 (pV197M) has been described to protect against COVID-19 in different populations because it is present in an exonic splicing enhancer site associated with protein malformation (45). This result also agrees with the study of Schönfelder et al. (2021), who also concluded that there is no association between rs12329760 and the risk of infection or COVID-19 severity (46).

In some studies, TMPRSS2 expression of the G allele of rs2070788 was associated with increased protein expression in lung tissue, which could lead to an association with increased susceptibility to COVID-19 (35, 41). Recently, a bioinformatics analysis performed by Mujalli et al. (2022) revealed the overexpression of genes implicated in the ACE2-TMPRSS2 signaling pathway in COVID-19 patients with severe and fatal phenotypes. Moreover, *SERPINE1* was identified as a drug target gene of *TMPRSS2*, with a similarity score of 0.54, which could suggest an interaction with the spike protein of SARS-CoV-2 (47).

The *SERPINE1* gene encodes PAI-1, which is implicated in coagulopathy and regulates the balance between coagulation and fibrinolytic systems. In some studies, coagulopathies have been reported in patients with severe COVID-19 (48). The mechanism of thrombosis in patients with COVID-19 may involve a cytokine syndrome that activates the coagulation process. For the imbalance of PAI-1, the coagulation process, among other factors, has been implicated as a genetic risk factor. SERPINE1 has been associated with thrombosis in diseases such as ischemic stroke, cancer, and, more recently, COVID-19 (10, 49–52).

In that sense, COVID-19 patients exhibit variable states of coagulopathy, with a marked thrombotic tendency among nonsurviving individuals. Salem N. et al. reported that 31% of COVID-19 patients exhibited increased hypercoagulability with hypofibrinolytic capacity (53, 54). These findings indicate the potential implications of *SERPINE1* genetic variants for COVID-19 outcomes. Moreover, in infectious diseases, PAI-1 plays a role in the inflammatory process as a mediator of the early host defense response to combat pathogens and inhibits fibrinolysis and could be related to thrombophilia (55).

The association of several *SERPINE1* polymorphisms with previously described pathologies has been reported and could represent a risk factor for severe COVID-19. The *SERPINE1* polymorphisms evaluated in this study included rs2227631 (-1844 G/A), which is located in the promoter region and has been implicated as a regulatory region variant with possible functional loci; however, functional studies are necessary to explore the specific effects on COVID-19 severity (18). Furthermore, rs2227667, rs2070682, and rs2227692, located in intronic regions, were also analyzed (14).

In the present study, we observed a greater D-dimer concentration (>500 ng/mL) in COVID-19 patients, similar to that reported by others from different populations, who reported higher D-dimer concentrations in patients with COVID-19 and critical illness (21, 56). Chocron et al. (2021) reported that D-dimer is one of the measures used to detect COVID-19 severity; increased D-dimer levels could be a risk marker of thrombotic events (21). However, other authors found no association between D-dimer levels and death in COVID-19 patients (57, 58).

In addition, it has been reported that in patients with COVID-19, hyperinflammation can induce dysfunction and damage in endothelial cells, resulting in increased D-dimer levels (59). Lange et al. (2008) reported that *SERPINE1* genetic variants were associated with D-dimer concentrations in older European and African-American populations and that rs2227667 was associated with higher D-dimer levels and fibrin deposits (11); however, gene SNPs explained ≈2% of the total variation in D-dimer levels (60).

Coagulation biomarkers such as D-dimer, which are associated with SERPINE1 genetic variants, could increase the risk of complications due to coagulation activity in patients with severe COVID-19. Identifying patients at risk of complications in clinical practice could improve treatment and outcomes to optimize health services. Lopez-Castaneda et al. (2021) suggested the use of low-molecular-weight heparin, as a prophylactic treatment for COVID-19 patients, to limit the hypercoagulable state (13).

Dittmann et al. (2015) showed that some *SERPINE1* SNPs could inhibit TMPRSS2, showing an antiviral effect against the influenza A virus (61). rs2227631 and rs2227667 of the *SERPINE1* gene could have antiviral effects by preventing viral membrane fusion of SARS-CoV-2, leading to the inhibition of TMPRSS2-mediated S protein cleavage. In this sense, Rosendal et al. (2022) showed that *SERPINE1* prevents the cleavage of the S protein by binding to *TMPRSS2* (5). Moreover, the authors found that the antiviral effects of *SERPINA1, SERPINE1, SERPINE2*, and *SERPINF1* were observed during the first steps of infection in HBEC ALI cultures, revealing reduced SARS-CoV-2 entry into target cells (5).

Treatment with serpentine targets could decrease lung inflammation and modify thrombotic protease and complement levels. PEGSerp-1 has been developed as a new anti-inflammatory therapeutic or biologic for vascular damage, coagulation disorders, and inflammation damage (1). The highlights of the present study contribute to the knowledge of the specific polymorphisms that could affect COVID-19 severity through the identification of new pharmacological targets and treatment strategies to block SARS-CoV-2 entry via TMPRSS2 inhibition, such as argatroban and famotidine, which act as new scaffolds for TMPRSS2 inhibition (62), and other future treatments that could be used for other similar diseases.

SERPINE has been identified as an endogenous antiviral molecule against SARS-CoV-2 and could represent a possible treatment option due to its biological role in inhibiting the entry of the virus into host cells (22). Polymorphisms of *TMPRSS2* and *SERPINE1* could be associated with COVID-19 severity, modifying the susceptibility to fatal outcomes. However, it is necessary to elucidate the genetic susceptibility to severe disease caused by SARS-CoV-2.

In response to the recent COVID-19 pandemic, the search for therapeutic targets to combat the severity and complications of infection caused by SARS-CoV-2 has led to the identification of SERPINE1 as a natural inhibitor of the TMPRSS2 protease that enhances the viral infection process.

**Figure 2:**
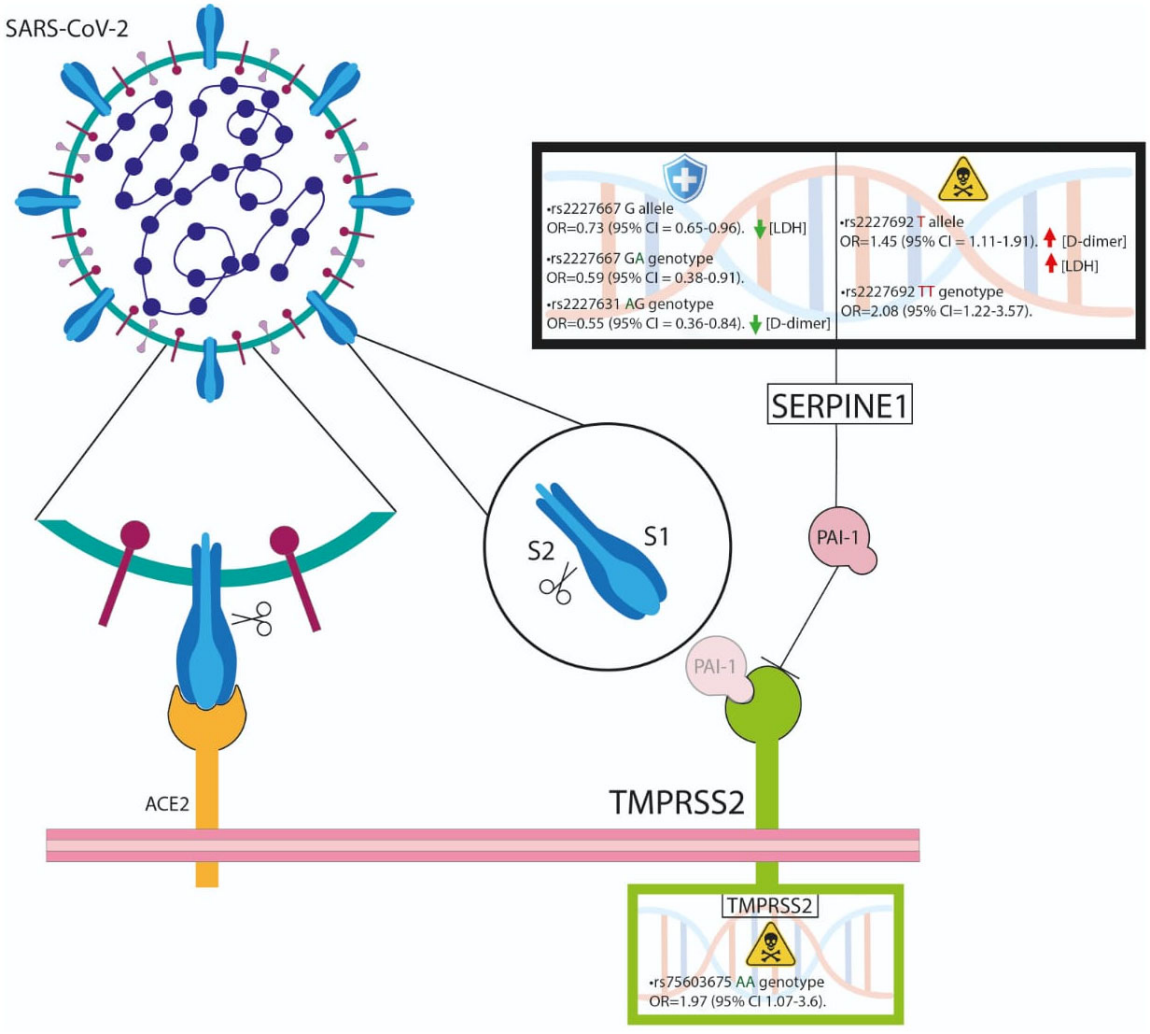
The *TMPRSS2* rs75603675 and *SERPINE1* rs2227692 variants are associated with poor outcomes. SERPINE1 could influence the altered processes of fibrinolysis development in hypercoagulable patients.

In conclusion, our study revealed that the *TMPRSS2* rs75603675 gene variant may induce an amino acid change from glycine to valine, which is more frequent in patients with COVID-19 who die. Similarly, we were able to demonstrate the association of the *SERPINE1* rs2227692 variant not only with a decreased outcome but also with an increase in D-dimer, which could influence the altered processes of fibrinolysis development in patients with a hypercoagulable state, as observed in critical pathology outcomes (**Figure 2**).

## Data availability statement

The original contributions presented in the study are included in the article/**Supplementary Material**, further inquiries can be directed to the corresponding authors.

## Ethics statement

The study was approved by the ethics committee of (INR-LGII: 17/20). The studies were conducted in accordance with the local legislation and institutional requirements. The participants provided their written informed consent to participate in this study.

## Author contributions

LM-G: Formal analysis, Methodology, Software, Writing – original draft. CM-A: Methodology, Writing – original draft. TT-L: Conceptualization, Supervision, Validation, Writing – review & editing. PV-C: Visualization, Writing – review & editing. RV-V: Resources, Writing – review & editing. JR-H: Data curation, Writing – review & editing. DG-M: Visualization, Writing – review & editing. GV-A: Data curation, Writing – review & editing. RP-S: Data curation, Writing – review & editing. JF: Data curation, Writing – review & editing. AP: Validation, Writing – review & editing. JR-P: Data curation, Investigation, Writing – review & editing. MM-M: Resources, Writing – review & editing. GV-Z: Resources, Writing – review & editing. AM-C: Resources, Writing – review & editing. FM-R: Resources, Writing – review & editing. DZ-A: Resources, Writing – review & editing. LR-T: Investigation, Resources, Writing – review & editing. AM-A: Visualization, Writing – review & editing. MC-R: Investigation, Resources, Writing – review & editing. MO-S: Methodology, Validation, Writing – review & editing. YS-K: Resources, Writing – review & editing. CS-A: Data curation, Writing – review & editing. JO-T: Investigation, Methodology, Writing – review & editing. BH-L: Investigation, Methodology, Writing – review & editing. CP: Project administration, Writing – review & editing. GM-N: Supervision, Writing – review & editing. AL-R: Funding acquisition, Project administration, Supervision, Writing – review & editing.

## Supplementary material

Supplementary 1
*TMPRSS2* and *SERPINE1* polymorphism allelic and genotype frequencies in the population study.
