# Supplementary material for: The fatal contribution of serine protease-related genetic variants to COVID-19 outcomes
Source: Front Immunol. 2024 Mar 27;15:1335963. doi: 10.3389/fimmu.2024.1335963 (PMC11004237; doi:10.3389/fimmu.2024.1335963)
Supplement: Supplementary 1 — TMPRSS2 and SERPINE1 polymorphism allelic and genotype frequencies in the population study. [file Table_1.docx]

Supplementary Material

# Supplementary Tables

**Table S1. *TMPRSS2* and *SERPINE1* polymorphisms allelic and genotype frequencies in population study.**

| Polymorphisms | Total  n = 1536 (100%) | Mild  n = 543 (35%) | Severe  n = 503 (33%) | Critical  n = 278 (18%) | Deceased  n = 212 (14%) | P value** | HWE |
| --- | --- | --- | --- | --- | --- | --- | --- |
| *TMPRSS2* | | | | | | | |
| *rs2070788* |  |  |  |  |  |  |  |
| G | 1682 (57%) | 556 (55%) | 580 (59%) | 338 (61%) | 208 (50%) | **0.003** |  |
| A | 1286 (43%) | 448 (45%) | 410 (41%) | 218 (39%) | 210 (50%) |  |  |
| GG | 478 (32%) | 152 (30%) | 165 (33%) | 106 (38%) | 55 (26%) | **0.009** | 0.78 |
| GA | 726 (49%) | 252 (50%) | 250 (50%) | 126 (45%) | 98 (47%) |  |  |
| AA | 280 (19%) | 98 (19%) | 80 (16%) | 46 (16%) | 56 (27%) |  |  |
| *rs75603675* |  |  |  |  |  |  |  |
| C | 2157 (73%) | 729 (73%) | 738 (75%) | 405 (75%) | 285 (68%) | **0.04** |  |
| A | 779 (27%) | 269 (27%) | 244 (25%) | 133 (25%) | 133 (32%) |  |  |
| CC | 826 (56%) | 276 (55%) | 290 (59%) | 158 (59%) | 102 (49%) | 0.23 | **0.03** |
| CA | 505 (34%) | 177 (35%) | 158 (32%) | 89 (33%) | 81 (39%) |  |  |
| AA | 137 (9%) | 46 (9%) | 43 (9%) | 22 (8%) | 26 (12%) |  |  |
| *rs12329760* |  |  |  |  |  |  |  |
| C | 2306 (86%) | 842 (85%) | 707 (87%) | 441 (86%) | 316 (83%) | 0.41 |  |
| T | 386 (14%) | 144 (15%) | 109 (13%) | 69 (14%) | 64 (17%) |  |  |
| CC | 994 (74%) | 360 (73%) | 308 (76%) | 194 (76%) | 132 (69%) | 0.62 | 0.85 |
| CT | 318 (24%) | 122 (25%) | 91 (22%) | 53 (21%) | 52 (27%) |  |  |
| TT | 34 (2%) | 11 (2%) | 9 (2%) | 8 (3%) | 6 (3%) |  |  |
| *SERPINE1* | | | | | | | |
| *rs2227631* |  |  |  |  |  |  |  |
| G | 1979 (75%) | 722 (73%) | 600 (75%) | 373 (74%) | 284 (77%) | 0.56 |  |
| A | 671 (25%) | 260 (26%) | 198 (25%) | 129 (26%) | 84 (23%) |  |  |
| GG | 756 (57%) | 271 (55.2%) | 226 (57%) | 142 (56%) | 117 (64%) | 0.27 | 0.20 |
| GA | 468 (35%) | 180 (37%) | 148 (37%) | 89 (35%) | 50 (27%) |  |  |
| AA | 102 (8%) | 40 (8%) | 25 (6%) | 20 (8%) | 17 (9%) |  |  |
| *rs2227667* |  |  |  |  |  |  |  |
| A | 1427 (55%) | 505 (54%) | 442 (55%) | 267 (53%) | 213 (58%) | 0.37 |  |
| G | 1173 (45%) | 427 (46%) | 356 (45%) | 239 (47%) | 151 (42%) |  |  |
| AA | 407 (31%) | 149 (32%) | 120 (31%) | 69 (27%) | 69 (38%) | 0.13 | **0.02** |
| AG | 613 (47%) | 207 (44%) | 202 (51%) | 129 (51%) | 75 (41%) |  |  |
| GG | 280 (21%) | 110 (24%) | 77 (19%) | 55 (22%) | 38 (21%) |  |  |
| *rs2070682* |  |  |  |  |  |  |  |
| T | 2018 (78%) | 733 (78%) | 622 (78%) | 388 (77%) | 275 (76%) | 0.78 |  |
| C | 578 (22%) | 201 (21%) | 174 (22%) | 118 (23%) | 85 (24%) |  |  |
| TT | 787 (61%) | 286 (61%) | 241 (60%) | 148 (58%) | 112 (62%) | 0.11 | 0.78 |
| TC | 444 (34%) | 161 (35%) | 140 (35%) | 92 (36%) | 51 (28%) |  |  |
| CC | 67 (5%) | 20 (4%) | 17 (4%) | 13 (5%) | 17 (9%) |  |  |
| *rs2227692* |  |  |  |  |  |  |  |
| C | 1376 (52%) | 512 (53%) | 416 (52%) | 274 (55%) | 174 (47%) | 0.18 |  |
| T | 1250 (48%) | 446 (46%) | 384 (48%) | 228 (45%) | 192 (52%) |  |  |
| CC | 380 (29%) | 147 (31%) | 113 (28%) | 77 (31%) | 43 (23%) | 0.52 | 0.07 |
| CT | 616 (47%) | 218 (45%) | 190 (47%) | 120 (48%) | 88 (48%) |  |  |
| TT | 317 (24%) | 114 (24%) | 97 (24%) | 54 (21%) | 52 (28%) |  |  |

**Chi-square. *Hardy Weinberg-Equilibrium (HWE) in mild outcome. Values in bold denotes statistical significance.
